# RNAi Mediated Hypoxia Stress Tolerance in Plants

**DOI:** 10.3390/ijms21249394

**Published:** 2020-12-10

**Authors:** Federico Betti, Maria José Ladera-Carmona, Pierdomenico Perata, Elena Loreti

**Affiliations:** 1 PlantLab, Institute of Life Sciences, Sant’Anna School of Advanced Studies, 56010 Pisa, Italy; f.betti@santannapisa.it (F.B.); Maria.Ladera-Carmona@agrar.uni-giessen.de (M.J.L.-C.); perata.pierdomenico@santannapisa.it (P.P.); 2 Institute of Agricultural Biology and Biotechnology, National Research Council, 56124 Pisa, Italy

**Keywords:** argonaute, hypoxia, miRNAs, RNAi

## Abstract

Small RNAs regulate various biological process involved in genome stability, development, and adaptive responses to biotic or abiotic stresses. Small RNAs include microRNAs (miRNAs) and small interfering RNAs (siRNAs). MicroRNAs (miRNAs) are regulators of gene expression that affect the transcriptional and post-transcriptional regulation in plants and animals through RNA interference (RNAi). miRNAs are endogenous small RNAs that originate from the processing of non-coding primary miRNA transcripts folding into hairpin-like structures. The mature miRNAs are incorporated into the RNA-induced silencing complex (RISC) and drive the Argonaute (AGO) proteins towards their mRNA targets. siRNAs are generated from a double-stranded RNA (dsRNA) of cellular or exogenous origin. siRNAs are also involved in the adaptive response to biotic or abiotic stresses. The response of plants to hypoxia includes a genome-wide transcription reprogramming. However, little is known about the involvement of RNA signaling in gene regulation under low oxygen availability. Interestingly, miRNAs have been shown to play a role in the responses to hypoxia in animals, and recent evidence suggests that hypoxia modulates the expression of various miRNAs in plant systems. In this review, we describe recent discoveries on the impact of RNAi on plant responses to hypoxic stress in plants.

## 1. Introduction

The discovery of microRNAs (miRNAs) as regulators of gene expression was a crucial step in understanding the transcriptional and post-transcriptional regulation in plants and animals. miRNAs are small endogenous RNAs with 21–24 nucleotides that originate from the processing of non-coding primary miRNA (pri-miRNA) transcripts folding into hairpin-like structures [1]. The mature miRNAs that derive from the pri-miRNA precursors are incorporated into the RNA-induced silencing complex (RISC) and drive the Argonaute (AGO) proteins towards the mRNA targets.

In plants, miRNAs interact with their targets through perfect or near perfect complementarity. The AGO proteins regulate the expression of miRNA target genes via epigenetic modification, mRNA cleavage or translational repression. Many miRNAs are highly conserved across plant species and are considered important gene regulators since the majority target transcription factors that participate in common biological processes, such as cellular differentiation, metabolism and hormone signaling.

Changes in miRNA expression levels thus have a strong impact on plant growth and development. In the last decade, miRNAs, whose numbering is sequential, have been found to regulate plant growth and development, as well as the response of plants to biotic and abiotic stresses [2,3,4]. In plants, several miRNAs are stress-regulated under nutrient deprivation, salinity, cold and UV-B radiation [3,4,5,6]. In addition, many miRNAs associated with plant growth show altered expression levels during stress, indicating that miRNAs regulate plant adaptation to stress conditions.

Hypoxic stress, mainly caused by flooding events, compromises the oxygen diffusion in plant tissues leading to cellular hypoxia, decreases in ATP production, and to a burst in the production of reactive oxygen species (ROS) [7]. Plant responses to hypoxia consist in a large reorganization of the transcriptome, inducing a shift from aerobic metabolism to anaerobic fermentation in order to satisfy the energy demand of the stressed cell [8,9].

In plants, hypoxia sensing occurs through the oxygen-dependent destabilization of the transcription factors belonging to the group VII Ethylene Responsive (ERFVIIs), with RELATED TO APETALA 2.2 (RAP2.2) and RAP2.12 playing a major role [10,11]. The oxidation of the conserved *N*-terminal Cysteine (Cys) residue which characterizes ERFVII is catalyzed by PLANT CYSTEINE OXIDASES (PCOs) [12,13] and targets ERFVII TFs for proteasomal degradation [14,15]. However, the lack of oxygen that occurs upon hypoxic conditions dampens the activity of PCOs leading to ERFVII stabilization. Under hypoxia, ERFVIIs translocate into the nucleus and bind a cis-regulatory motif, the hypoxia-responsive promoter element (HRPE), in order to regulate the transcription of the hypoxia-responsive genes [16].

Among the hundreds of genes that show altered expression levels upon hypoxia in Arabidopsis (*Arabidopsis thaliana*), 49 genes are ubiquitously expressed in all organs and cell types in response to low oxygen [17]. These 49 hypoxia-responsive genes (HRGs) constitute the core of the anaerobic response in plants, and are efficiently and quickly transcribed, polyadenylated and translated upon low oxygen concentrations [18,19]. On the other hand, genes associated with growth and major cellular processes, including ribosomal protein genes (RPs) are constantly transcribed and polyadenylated; however, nuclear export and translation of their mRNAs are dampened until the tissues have been re-aerated [19,20].

Transcriptomic analyses have highlighted changes in the abundance of mature miRNAs and their precursors in Arabidopsis and corn roots under oxygen deprivation [11,21,22]. Interestingly most of these miRNAs target genes encoding for transcription factors associated with plant growth and tissue differentiation. Since upregulation of miRNAs reduces the abundance of target mRNAs, hypoxia-upregulated miRNAs may repress the negative regulators of the hypoxic response or slow down plant growth during the stress. Conversely, the downregulation of miRNAs increases the abundance of their target mRNAs, which may help in adapting to stress.

Although knowledge on the plurality of gene regulatory networks activated by hypoxia has increased considerably in the last few years, our understanding of the role of RNA silencing upon hypoxia is still limited and the function of hypoxia-responsive miRNAs remains elusive. Here, we summarize recent work on the regulatory roles of miRNAs in the adaptative responses of plants to low oxygen conditions.

## 2. Post-Transcriptional Gene Silencing

The modulation of gene expression also occurs beyond the classical transcriptional regulation, through the activity of transcriptional factors (TFs) binding to gene promoters and includes Transcriptional Gene Silencing (TGS) and Post-Transcriptional Gene Silencing (PTGS). In TGS transcription is repressed, while in PTGS the translation does not occur because the messenger RNA (mRNA) is degraded or the translation is inhibited.

mRNA translation is prevented by mRNA cleavage or via translational repression, both processes mediated by small RNAs (smRNAs). smRNAs are composed of between 20 to 27 nucleotides, which do not encode for a protein, and based on their biogenesis, they are divided in two main groups: micro RNAs (miRNAs) and small interference RNA (siRNAs). While miRNAs are transcribed from genes that are different from those that they target, siRNAs have a different biogenesis, and originate from the processing of a long double-stranded RNA (dsRNA) or from a single stranded RNA molecule forming a hairpin. In this latter case, many siRNAs species can be generated from the same hairpin [23].

It has also been demonstrated that in *Caenorhabditis elegans* and in plants, miRNAs and siRNA are cell-to-cell mobile. This means that they can regulate target genes in different cells from the ones where they were produced [24]. This trafficking may occur from cell to cell via plasmodesmata or in more distant parts of the plant via phloem [25,26]. During submergence, Arabidopsis plants overexpressing miR399 show a strong repression of the target PHOSPHATE 2 (PHO2) [27], indicating that PTGS is able to operate under hypoxia [28].

## 3. miRNAs Biogenesis

miRNAs are small noncoding RNAs of 21–24 nt that determine post-transcriptional gene regulation via mRNA cleavage or the repression of mRNA translation [1,29]. miRNAs are transcribed by RNA Polymerase II (Pol II) from the nuclear-encoded miRNA genes (MIR) [30,31]. In plants, MIR genes are located in intergenic regions of the genome and encode for independent transcription units [32]. The overrepresentation of TATA boxes and cis-regulatory motifs in miRNA promoters suggests an involvement of many trans-acting factors in the regulation of MIR expression [29], as expected for genes that are transcribed only during specific moments of plant growth or in response to specific stimuli. The resulting transcript called primary RNA (pri-miRNA) folds into an intramolecular hairpin structure containing imperfectly base-paired segments. The addition of a 5′-methylguanosine cap [33] and a 3′ polyadenylate tail [34,35] stabilizes the pri-miRNA.

miRNAs are released from pri-miRNA through two sequential cleavage events catalysed by the RNA helicase DICER-LIKE 1 (DCL1) [36,37]. In Arabidopsis, the correct biogenesis of miRNAs requires an interaction between DCL1, the RNA-binding protein DAWDLE (DDL), the zinc-finger SERRATE (SE) [38,39], a dsRNA binding protein HYPONASTIC LEAVES (HYL1) [40,41] and components of the nuclear capping complex (CBC) and of the splicing machinery [1].

The first cleavage event occurs near the base of the stem and generates the precursor miRNA (pre-miRNA), which maintains a stem-loop structure. Subsequently, a second cleavage event converts the pre-miRNA hairpin precursor into a shorter double-stranded RNA duplex (miRNA::miRNA*) consisting of the mature guide strand (miRNA) and the passenger strand (miRNA*) [41,42]. In plants, the subsequent DCL1 cleavage event occurs progressively in the nucleus at 21 nucleotide intervals along the stem [29]. After DCL1 processing, the miRNA::miRNA* duplex is stabilized and protected from degradation via 2′-0-methylation of the 3′ terminus of each strand mediated by HUA ENHANCER 1 (HEN1) [43,44]. Loss of function mutants *hen1* and *dcl1* leads to a lower accumulation of most miRNAs species than wild type plants, confirming the need for functional DCL1 and HEN1 proteins in miRNA biogenesis [45]. The methylated miRNA::miRNA* duplex is then exported from the nucleus to the cytoplasm by HASTY (HST) [46], the *Arabidopsis* homolog of exportin 5. In the cytoplasm, the mature-miRNA strand is separated from the passenger strand. It is then incorporated into ARGONAUTE proteins (AGOs), which constitute the catalytic component of the RNA-induced silencing complex (RISC).

In plants, the correct RISC assembly requires other cytosolic proteins, such as SQUINT (SQN) and HEAT SHOCK PROTEIN90 (HSP90) [47,48]. The RISC complex recognizes the target transcript through sequence specific base-pairing between the miRNA and the target RNA. In this process, miRNA acts only as a guide to direct the AGO protein to the correct target [1]. Depending on the level and position of the complementarities between the guide strand and the mRNA target, miRNAs induce mRNA cleavage or repress their translation [49,50].

Target slicing requires a high degree of sequence complementarity between the miRNA and the target sequence [29], while translation inhibition can also still happen with a lower degree of complementarity [51]. Because of the extensive sequence complementary between plant miRNAs and their targets, mRNA-slicing has been reported as the main pathway occurring in plants [1]. In addition to post transcriptional gene silencing (PTGS), plant miRNAs can also affect gene transcription, promoting cytosine methylation via sequence-specific targeting of chromatin [52,53]. Transcription factors (TFs) and stress responsive proteins are the major targets of miRNAs, suggesting that miRNAs have important roles in plant development, organ differentiation, nutrient homeostasis, and responses to biotic and abiotic stresses [3,4].

## 4. Hypoxia Affects the Expression of miRNAs Associated with Plant Growth

Flooding is a compound stress characterized by limited underwater gas diffusion and low light levels in turbid waters. Under these conditions, oxidative phosphorylation and photosynthesis are drastically compromised, and plants are subjected to energy and carbohydrate starvation crisis. Plants respond to this energy crisis with a large reorganization of the transcriptome, which promotes a shift from aerobic metabolism to anaerobic fermentation [9,54,55,56]. However, anaerobic fermentation, coupled with starch degradation, constitutes a valid alternative to aerobic respiration only during short and transient hypoxia, while it is not able to satisfy the plant energy demand under prolonged hypoxia [57].

Two of the strategies developed by plants in response to flooding are the low-oxygen escape strategy (LOES) and the low-oxygen quiescence strategy (LOQS) [54,57]. In the escape strategy, the plant develops phenotypic traits that increase the surface area of gas exchange between the plant tissues and the atmosphere in order to reestablish a good supply of oxygen. Escape phenotypes include shoot elongation, hyponastic leaves, aerenchyma formation, and the development of lateral and adventitious roots [54,57]. In contrast to LOES, LOQS consists in the cessation of plant growth and management of metabolism which delay the energy crisis and redirect plant growth when normoxia resumes. Plants that adopt a quiescence strategy reduce the investment of energy in processes associated with cell division and growth, such as DNA, protein, ribosome and cell wall synthesis [10,57].

Both upregulation and downregulation have been observed of different miRNAs in Arabidopsis and maize roots during oxygen deprivation [11,21,28]. Moldovan et al. [21] found that the abundance of 25 miRNAs was altered in Arabidopsis roots upon hypoxia. MiR156g, miR157a, miR158a, miR159a, miR172a, b, miR391 and miR775 were upregulated after 5 h of hypoxic treatment. As is characteristic of miRNAs, many targets of the hypoxia-responsive miRNAs identified by Moldovan et al. [21] are transcription factors belonging to the MYB, Homeobox, SQUAMOSA PROMOTER BINDING PROTEIN LIKE (SPL), AUXIN RESPONSIVE FACTOR (ARF), APETALA2 (AP2), MADS and CCAT-HAP2 families [21]. miRNAs may therefore be implicated in the regulation of plant growth and cell differentiation under oxygen deprivation.

In Arabidopsis, miR156 and miR172 regulate juvenile-adult transition by repressing the expression of their target genes, SPL and AP2, respectively [58,59]. SPL and AP2 proteins have been reported to play a central role in plant growth and floral development [59]. Single and double mutants for SPL9 and SPL15 show a shortened vegetative plastochron and enhanced branching, all characteristics that have already been observed in plants overexpressing miR156b [60]. Therefore, miR156 promotes the juvenile phase regulating SPL genes via cleavage or translation repression. On the other hand, miR172 targets AP2 transcripts in order to promote flowering and adult epidermal identity [58,61]. The double mutant for TOE1 and TOE2, two of the main AP2 proteins, flowers much earlier than wild type plants, while the overexpression of TOE1 causes a delay in the flowering [61]. Alteration in the expression level of miR156 and miR172 may therefore control or arrest plant growth upon low oxygen concentration.

As mentioned above, the production of lateral and adventitious roots is another typical response adopted by plants to deal with the reduction in oxygen diffusion in the rhizosphere. In Arabidopsis, many miRNAs that are implicated in the regulation of root growth and architecture show altered expression upon oxygen deprivation [62]. For instance, miR166/165 promote primary root elongation and maintain the root apical meristem [63,64]. miR394 is involved in the maintenance of embryonic shoot apical meristems [65]. miR390 regulates lateral root formation [66]. miR160 and miR167 are involved in adventitious root development [67,68]. In Arabidopsis, miR167 cleaves the ARF transcripts.

ARFs are a large family of DNA-binding proteins that regulate the expression of auxin-responsive genes and the lateral root formation. miR167 was found to be upregulated at the level of the roots of maize and rice in the early phase of submergence and in Arabidopsis roots during hypoxia [21,69]. However, the same miRNA was downregulated in maize seedlings during waterlogging [22]. This discrepancy suggests that miRNAs expression levels may vary in response to stress severity and plant growth conditions. Kinoshita et al. (2012) reported that miR167 cleaves the transcript of IAA-Ala Resistant 3 (IAR3).

In Arabidopsis, IAR3 participates in root development by catalyzing the conversion of an inactive form of auxin into the bioactive form. Interestingly, osmotic stress drastically reduces the abundance of miR167 and increases the expression level of IAR3 leading to the formation of lateral roots [70]. The active role of miR167-IAR3 interaction in the regulation of the root architecture was supported by the absence of lateral roots in the iar 3 mutant upon osmotic stress [70]. Since auxin has a central role in root growth [71], alteration in miR167 abundance may affect root architecture, modulating the expression level of different targets upon stress conditions.

Of the 25 hypoxia-responsive miRNAs identified by Moldovan et al. (2010), miR166 is one of the few miRNAs with reduced abundance in Arabidopsis roots upon low oxygen concentration. miR166 cleaves the HD-ZIP III transcript and negatively regulates the lateral root development [63]. In Arabidopsis and *Medicago truncatula*, the overexpression of miR166 enhances primary root length and decreases lateral root formation [63,72]. On the other hand, the overexpression of HD-ZIP III inhibits root growth and reduces the root apical meristem activity. The down-regulation of miR166 in response to low oxygen concentration may therefore be required for lateral root formation.

The above examples suggest that miRNAs are involved in the adaptive responses of plants to low oxygen concentration. An alteration in miRNA abundance may regulate the plant growth and changes in the root architecture, which are typical responses adopted by plants to the energy crisis caused by the block of mitochondrial activity and reduce the distance from the aerial surface and the flooded tissues [54,57]. Interestingly, chemical inactivation of mitochondrial respiration was found to alter the expression of some hypoxia-responsive miRNAs, suggesting that miRNA regulation under hypoxia may be linked to mitochondrial functionality [21,28].

## 5. ROSs Modulate Expression of miRNAs during Hypoxia

The increase in reactive oxygen species (ROSs) or oxidative stress is a consequence of various environmental stresses, including hypoxia. In hypoxic tissues, enhanced ROS generation is due to altered mitochondrial functionality and has detrimental effects on macromolecules and organelles of the cell [7]. Cellular components that exhibit high ROS susceptibility include lipids, proteins and nucleic acids. In particular, lipid peroxidation and alteration of the membrane integrity cause dehydration and cell death. The susceptibility to hypoxia-induced oxidative stress changes between tissues and species, and is the consequence of several components, such as membrane composition, endogenous antioxidant content, and the ability to activate a timely antioxidant response.

One of the first responses of plants to oxidative stress is the activation of the superoxide dismutases (SODs). SODs protect the cell from oxidative stress by converting the superoxide to hydrogen peroxide (H_2_O_2_) and molecular oxygen [73,74]. In Arabidopsis, the expression of two Cu/Zn superoxide dismutases (CSD1 and CSD2) is enhanced in response to oxidative stress [73]. Sunkar et al. [5] demonstrated that CSD1 and CSD2 transcripts are targets of miR398. The downregulation of miR398 under oxidative stress is important for the posttranscriptional regulation of CSD1 and CSD2 [75]. Arabidopsis plants overexpressing a miR398-resistent form of CSD2 accumulate more CSD2 transcripts than plants overexpressing the wild type sequence and show less susceptibility to high light, heavy metals, and oxidative stresses [75].

Loreti et al. [28] reported the repression of miR398 in Arabidopsis adult plants under submergence. miR398 was also repressed by inhibitors of mitochondrial functionality, suggesting that mitochondrial respiration plays a central role in the regulation of miRNA expression under submergence [21,28]. However, the authors observed no changes in the expression of CSD1 and CSD2, either at the mRNA or at the protein level. Given that Sunkar et al. [5] showed that mir398 and its targets are differently expressed between plant tissues, the repression of miR398 and induction of CSD1 and CSD2 in response to hypoxia may only be observed in specific tissues of the plant. Alternatively, the upregulation of CSD1 and CSD2 may be required only later to respond to the burst of ROS occurring during the submergence recovery and reaeration of the tissues. Expression of miR398 is also affected by the ERF-VII transcription factor. miR398 is already downregulated under normoxia in plants overexpressing a stable form of the oxygen sensor RAP2.12, suggesting that oxygen sensing and mitochondrial activity may converge in the regulation of miRNA expression [28].

The abundance of several miRNAs has been found to be altered in rice seedlings after short-term exposure to H_2_O_2_. In particular, miR169, miR397, miR827 and miR1425 were upregulated while miR528 was downregulated by oxidative stress [76]. The putative targets of the identified H_2_O_2_-responsive miRNAs are involved in various biological processes such as nutrient homeostasis, IAA signaling, growth and cell differentiation. For instance, miR169 is upregulated by drought and salt stress [35,77]. miR169 targets the HAP2 subunits of the trimeric transcription factor complex NF-Y which is involved in the regulation of cell differentiation and mitochondrial respiration. miR169-mediated downregulation of HAP2 may therefore slow down processes associated with plant growth and development during stress.

miR397 represses the expression of laccases and plantacyanin during diverse abiotic stresses and Cu starvation [76]. Upregulation of miR397 during stress conditions may contribute to Cu homeostasis, limiting non-essential biological processes and saving copper for antioxidant defense, as in SODs [5]. In addition, miR827 was found to regulate nutrient homeostasis, regulating the expression of the SPX protein [76]. The rice-specific miR1425 downregulates the transcripts of pentatricopeptide repeat (PPR) genes [78,79].

PPRs are a large family of RNA-binding proteins that are widely conserved in higher plants [80]. PPRs participate in many steps of plastid gene expression, such as transcription, splicing, editing, processing and translation. The induction of miR1425 and repression of PPRs may slow mitochondrial activity and cell division during abiotic stresses. In Arabidopsis, PPRs are targets of the TAS1 family of trans-acting-siRNAs (tasiRNAs) [21].

tasiRNAs are a specialized class of endogenous small RNAs which are generated by miRNA processing of the TAS gene transcripts and, similarly to miRNAs, they regulate the expression of their targets at the post transcriptional level [1,39]. Arabidopsis has four families of TAS genes (TAS1, TAS2, TAS3 and TAS4) and are transcribed by RNA polymerase II. tasiRNA synthesis requires a functional miRNA, which recognizes and triggers the cleavage of the non-coding RNA of TAR genes; the conversion of the cleaved RNA into a double strand RNA by RNA DEPENDENT RNA POLYMERASE 6 (RDR6), and the DCL4-mediated processing of the double-strand RNA in mature tasiRNAs [1,33,81]. TAS1/TAS2 and TAS4 transcripts are recognized by miR173 and miR828 respectively, and tasiRNAs are generated from the miRNA-cleaved 3′ fragments [82]. By contrast, miR390 triggers the cleavage of TAS3 precursor and the production of tasiRNAs from the 5′-cleavage fragment [82].

While miR173 and miR828 promote the synthesis of tasiRNAs via AGO1, miR390 has been found to be associated with AGO7 [1]. The abundance of tasiRNAs belonging to the TAS1 family increased in Arabidopsis roots under hypoxia [21]. The downregulation of the PPR genes via miRNAs and tasiRNAs-mediated silencing may protect mitochondrial from oxidative stress, and control plant growth and development during hypoxia.

TAS3 is another family of tasiRNAs that was found to be upregulated in Arabidopsis roots by hypoxia and mitochondrial inactivation [21]. TAS3 regulates the expression of ARF gene transcripts which are involved in auxin-signaling [66]. The induction of TAS3 during hypoxia may therefore be implicated in the regulation of plant growth and root architecture during hypoxia.

Hypoxia and the consequent oxidative stress modulate the expression levels of several miRNAs and tasiRNAs in various plant species. The targets of these two classes of small RNAs regulate a large range of biological processes, such as nutrient homeostasis, auxin signaling and antioxidant defense. Alteration in the expression levels of miRNAs and tasiRNAs may therefore be involved in controlling the developmental changes that occur in plants during and after hypoxia. In addition, the identification of hypoxia-responsive miRNAs, such as mir398, regulating the expression of enzymes involved in ROS scavenging, suggests that RNA-silencing may participate in the regulation of plant oxidative defenses. Given that hypoxia hampers mitochondrial functionality and enhances ROS production, mitochondrial respiration may be a major factor in smRNA regulation under hypoxia.

## 6. The Role of ARGONAUTE Proteins in the Plant Response to Hypoxia

ARGONAUTE proteins (AGOs) play a central role in the final executive step of all RNA-silencing processes. AGO proteins constitute the catalytic core of the RISC complex, and are defined by three highly-conserved domains: PAZ, MID and PIWI [83]. X-ray crystallography analysis of the prokaryotic *Thermus thermophilus* AGO protein revealed that AGOs fold into a bilobal structure creating a central pocket for sRNA binding [83]. The MID and PAZ domains are recognized as part of the miRNA sequence and anchor the 5′ and 3′-terminal end, respectively, while the PIWI domain assumes an RNase-H like fold structure with endonuclease activity mediated by the Asp-Asp-His (DDH) catalytic triad [1,84]. Together the PAZ, MID and PIWI domains contribute to the correct placement of the sRNAs in the AGO proteins and to the interaction between the sRNA and the respective RNA targets.

Although AGO proteins constitute an evolutionarily conserved protein family, the number of genes encoding for them varies greatly among organisms. The genome of *Schizosaccharomyces pombe* encodes only 1 AGO protein, while *C. elegans*, *Drosophila melanogaster* and humans express 27, 5 and 8 AGOs, respectively [83,85]. The Arabidopsis genome encodes for 10 AGO proteins which are classified into three major phylogenetic clades. The classification of plant AGOs is purely based on protein similarity, and therefore members of the same clade can exhibit completely different functions and may be associated with different RNA-silencing pathways.

AGO1, AGO5 and AGO10 constitute the first clade. They bind 21 nt small RNAs (miRNAs and siRNAs) and take place mainly in PTGS. AGO1 is the main effector of RNA-silencing and its role in PTGS has been widely studied in Arabidopsis [51,84]. A loss of function of AGO1 causes severe developmental abnormalities and plant sterility. However, fertile hypomorphic *ago1* mutants that are completely defective in PTGS have been identified, indicating that AGO1 is implicated both in RNA-silencing as well as in plant development and growth. AGO10 is the closest AGO1 homologous in Arabidopsis and is required for the maintenance of the shoot apical meristem (SAM). Expression of AGO10 in the adaxial domain of leaves reduces the level of miR165/166, leading to the stabilization and accumulation of the HD-ZIP III transcription factor required for the SAM maintenance as well as for the establishment of leaf adaxial-abaxial polarity [78]. Recent studies reported that the competition between AGO1 and AGO10 for miR165/166 plays a central role in SAM establishment.

The third member of this first clade is AGO5. Arabidopsis *ago5* mutants show defects in megagametogenesis, suggesting that AGO5 is required in male gametophyte development [1]. The second clade of Arabidopsis AGO proteins includes AGO2, AGO3 and AGO7. AGO2 is induced by DNA double-strand breaks and is required for the assembly of the repair foci. AGO2 also takes part in the plant antiviral response, as highlighted by the hypersusceptible of the Arabidopsis *ago2* mutants to viral infections [86,87,88]. AGO7 is a specialized Argonaute that binds miR390 almost exclusively to produce TAS3 family tasiRNAs implicated in the regulation of the auxin-signaling pathway and in the juvenile-to-adult phase transition [89,90]. The last member, AGO3, is derived from a recent duplication event at the AGO2 locus, however the role(s) and the mode(s) of action of AGO3 remain elusive. Finally, AGO4, AGO6, AGO8 and AGO9 belong to the third clade of Arabidopsis Argonaute proteins. AGO4 mainly associates with 24 nt siRNAs and is required for TGS by RNA-dependent DNA methylation (RdDM) [91]. AGO4 is required both for de novo and maintenance of DNA methylation of genomic loci, including genes and transposons. As with AGO4, AGO6 and AGO9 are also implicated in RdDM, but their expression from AGO4 promoter partially compensates for the loss of function of AGO4 in the *ago4* knockout mutant, suggesting that AGO6 and AGO9 mediate RdDM only at specific loci and in specific tissues. AGO8 is instead considered a pseudogene and therefore no functions have been attributed to this Argonaute.

The study of the role of RNA-silencing in response to hypoxia and flooding has led to the identification of several hypoxia-responsive miRNAs, while our knowledge of the effects of oxygen depletion on the transcription, localization and activity of AGO proteins remains limited. Recently [28] reported that Arabidopsis AGO1 and AGO4 may play a central role in the regulation of hypoxia-responsive genes and plant tolerance to submergence. The arabidopsis *ago1-27* mutant was found to be more sensitive to submergence than wild type plants. A microarray analysis revealed that activation of the core anaerobic genes in response to submergence is not altered in the *ago1-27* mutant, indicating that the expression of these genes is not mediated by RNA silencing [28]. However, the authors identified two groups of genes, which are not classified as core anaerobic genes, that were up- and down-regulated differently during submergence in wild type and *ago1-27* mutant plants.

It is therefore tempting to speculate that the activation of some submergence-related genes requires a functional AGO1 protein and a step involving PTGS or TGS. Interestingly, Liu et al. (2018) demonstrated that AGO1 associates with chromatin and facilitates the recruitment of RNAP II at specific loci in order to activate gene expression. The binding of AGO1 to specific gene loci was demonstrated to be mediated by the 21 nt sRNAs generated by DCL-1. The authors also reported that the association of AGO1 with chromatin was responsive to hormonal, biotic and abiotic stimuli, suggesting that the bound of AGO1 to gene loci could be an alternative way to PTGS through which AGO1 regulates gene expression under stress.

The state of the chromatin and epigenetic modifications are two other factors that significantly affect gene transcription. The methylation of specific cytosines as well as the post-translational modifications of histones can increase or reduce the accessibility on gene regions. Studies on histone modification dynamics have shown how epigenetic modifications contribute to the regulation of HRG activation [18,20]. In Arabidopsis, the coding sequence of hypoxia-responsive genes is enriched in nucleosomes containing the repressive Histone 2A variant H2A.Z which makes the chromatin less accessible to the transcriptional complex. Under hypoxic stress, the levels of the core H2A.Z subunit of nucleosomes decrease in HRGs in concomitance with their transcriptional activation. Hypoxia also significantly increases Histone 3 (H3) modifications associated with active transcription, including H3 Lysine 4 trimethylation (H3L4me3) and H3 Lysine 9 acetylation (H3L9ac).

In plants, RNA-directed DNA methylation (RdDM) is another RNA-silencing pathway that regulates gene expression via DNA methylation. RdDM is mediated by 24-nt siRNAs and requires a functional AGO4 protein [92,93,94]. Briefly, in Arabidopsis, Pol IV transcribes a short single-strand RNA of 30–40 nucleotides which is converted into a double-strand RNA by the RNA-DEPENDENT RNA POLYMERASE 2 (RDR2) and processed in 24-nt siRNA by DCL3. These siRNAs are loaded onto AGO4 and target a second set of nascent transcripts generated by Pol V. Ultimately, AGO4 promotes the de novo DNA methylation of cytosines at specific loci through the recruitment of chromatin modifiers such as the DOMAINS REARRANGED METHYLTRANSFERASE 1/2 (DRM1/2).

Alternative non-canonical RdDM that include steps catalyzed by RDR6, DCL2 and DCL4 appear to regulate the methylation status of chromatin [95]. However, the functions and the roles of the non-canonical RdDM variants are still not understood nor characterized. AGO4 was found to influence the expression of *HOMOLOG OF RPW8 4* (*HR4*), which is a gene encoding for a biotic-stress related protein [28]. The second exon of *HR4* gene is highly methylated in wild type plants, most likely because of its proximity to a transposable element located downstream of the gene. Infection of Pseudomonas syringae causes de-methylation of *HR4* and increases the transcription of the gene [96]. Interestingly, HR4 was also found to be up-regulated in wild type plants during prolonged submergence and in plants that constitutively express a stable version of RAP2.12 (*35S:∆-RAP2.12*), a transcription regulator of the hypoxia response [28]. In *ago4-1*, as well as in *35S:∆-RAP2.12* plants, the regional methylation of *HR4* is lower than in wild type plants, highlighting the existence of a link between the AGO4-mediated RdDM and the response to hypoxia driven by the ERF-VII TFs.

Zhang et al. [97] focused on RdDM in relation to the Arabidopsis response to salinity stress. They reported that AGO3 is barely expressed in normal growth conditions, however it can be strongly up-regulated by salinity stress, especially in roots. Since AGO3 binds both 21 and 24-nt siRNAs, they hypothesized that AGO3 is redundant to AGO4 in the epigenetic pathway. However, expression of AGO3 from the AGO4 promoter only partially rescues the hypomethylation in the *ago4-1* mutant, suggesting that AGO3 either operates only when it is significantly expressed by abiotic stresses such as salt stress [97] or plays a different role in the pathway. Notably, a study conducted in Arabidopsis reproductive tissues supports the hypothesis that AGO3 act via PTGS since it localizes both in the nucleus and cytoplasm of the cell [98,99]. A transcriptomic analysis performed using seedlings of *35S:∆-RAP2.12* [100] showed a strong up-regulation of AGO3 while none of the other genes involved in RNA silencing showed altered expression.

The strong expression of AGO3 because of the overexpression of a stable version of RAP2.12 in air suggests that ERFVII proteins may be required for the induction of AGO3, which could represent important evidence of a cross-talk between oxygen signaling and RNA silencing pathways. We scanned the first 1000 nucleotides upstream of the ATG start codon of the AGO3 gene using the MEME FIMO tool (Find Individual Motif Occurrences) to find the HRPE motif already reported to be bound by RAP2.2 and RAP2.12. The output of the analysis revealed the presence of the HRPE motif above 600 nucleotides upstream of the ATG codon, suggesting that expression of AGO3 could be activated via ERF-TFs. However further analyses are still needed to determine the hypothetical role of AGO3 in hypoxia response and plant tolerance to submergence.

## 7. Conclusions and Future Prospects

In eucaryotes, the regulation of protein-coding genes is highly dynamic and is controlled at different levels both in the nucleus and in the cytosol of the cell. Recent multiomic studies on chromatin dynamics, the cis-regulatory motif, RNAPII phosphorylation profile, alternative polyadenylation and polyadenylated ribosome-associated RNA profiling have highlighted how hypoxia regulates gene expression at multiple levels. Hypoxia leads to a large reorganization of the transcriptome, promoting metabolic and morphological changes in order to increase the tolerance and survival rate of the plant to stress.

The basis of plant responses to hypoxia involves the activation of the core set of 49 HRGs, which includes genes encoding key enzymes involved in anaerobic fermentation, antioxidant response and sucrose catabolism. The HRGs constitute a cluster of genes that are efficiently and quickly transcribed, polyadenylated and translated upon low oxygen concentrations. In addition to the HRGs, hypoxia regulates the expression of other genes that are associated with several pathways, including plant growth, cell differentiation, hormone signaling, heat and oxidative stress. These genes differ from HRGs in terms of epigenetic signature, transcriptional and translational time, and mRNA localization. Genes that encode proteins associated with heat stress, oxidative stress and reactive oxygen species signaling are progressively up-regulated upon hypoxic stress. This group of genes is transcribed in the early stages of hypoxia but the newly synthesized mRNAs accumulate in the cell nucleus and are not polyadenylated.

The translation of these mRNAs occurs only after prolonged hypoxia or reoxygenation, indicating that their products constitute a protective barrier against conditions associated with high levels of ROS production. In contrast, genes associated with growth and major cellular processes, including ribosomal protein genes (RPs), constitute a third cluster characterized by another mode of hypoxia regulation. RP genes are constantly transcribed by RNAPII and their transcripts are polyadenylated even upon hypoxic stress; however, nuclear export and translation of their mRNAs are dampened until re-aeration of the tissues. The translation of the existing cytosolic RP transcripts is also blocked through the sequestration of the RP mRNAs in protein-RNA complexes until the stress ends.

In plants and animals, the abundance and translation of mRNAs can be regulated via RNA silencing, as well as by TGS and PTGS. Together with Argonaute proteins, miRNAs are key components of the silencing machinery, and act as critical post-transcriptional modulators controlling the expression of their targets.

Many studies have been carried out in the last decade to clarify the role of RNA-silencing in gene regulation under hypoxia in plants. Submerged Arabidopsis plants overexpressing miR399 show a strong repression of the target PHO2 [27], indicating that PTGS is able to operate under hypoxic conditions [28]. The analysis of mutants for genes encoding proteins associated with different RNA-silencing pathways revealed that AGO1 and AGO4 are involved in the transcriptional regulation of hypoxia-responsive genes and in the plant tolerance to submergence. Arabidopsis *ago1-27* mutants are more susceptible to submergence than wild type plants, while AGO4 has been hypothesized to influence gene expression under hypoxia via RdDM. In addition, transcriptomic analysis highlighted changes in the abundance of mature miRNAs and their precursors in response to oxygen deprivation.

Unfortunately, it has not always been possible to establish a clear relationship between the expression of hypoxia-responsive miRNAs and the expression of their putative targets. Furthermore, miRNAs abundance varies considerably depending on the hypoxic conditions, suggesting that miRNA expression may vary between plant tissues and experimental conditions.

Although several hypoxia-related miRNAs have been identified, their role in plant response to hypoxia still needs to be clarified. Since many hypoxia-responsive miRNAs are associated with plant development, it is possible that miRNAs constitute a link between environmental factors and developmental modulation under hypoxic conditions. Up- and down-regulation may therefore increase the plant’s tolerance to hypoxia, redirecting plant growth when normoxia resumes.

**Figure 1:**
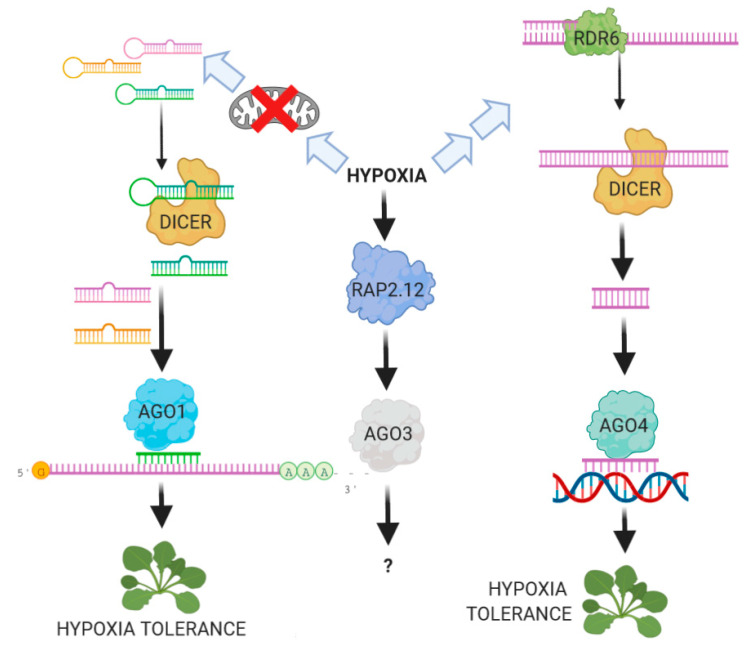
Influence of hypoxia on the RNAi machinery in plants. Under hypoxia mitochondrial activity is largely prevented due to the lack of oxygen. This is likely to be one of the factors triggering the production of some hypoxia-related miRNAs which, by relying on the activity of AGO1, contribute to hypoxia tolerance. Hypoxia tolerance is also influenced by AGO4, which controls RNA-dependent DNA-methylation pathways. The stabilization of the transcription factor RAP2.12 activates the transcription of AGO3, whose role in RNAi is still largely unknown.

The examples reported in this paper demonstrate that the adaptive response of plants to hypoxic stress is an intricate network of multiple factors which makes it complicated to isolate individual molecular pathways. In this context, miRNAs may play a central role in helping the plant to reach a physiological balance (Figure 1). Understanding the molecular mechanism of miRNA action during hypoxia and identifying the bases of the cross-talk between RNA-silencing and oxygen sensing may provide an efficient tool to help us promote the plant’s efficiency in responding to the flooding events caused by changes in the environment.

## Abbreviations

AGO	Argonaute
ERFVII	Group VII of Ethylene Responsive Factor
HRG	Hypoxia Regulated Gene
miRNA	Micro RNA
PTGS	Post-transcriptional Gene Silencing
RISC	RNA-induced silencing complex
siRNA	Small Interfering RNA
smRNA	Small RNA
TGS	Transcriptional Gene Silencing
